# Transcriptomics and metabolomics reveal changes in the regulatory mechanisms of osteosarcoma under different culture methods in vitro

**DOI:** 10.1186/s12920-022-01419-1

**Published:** 2022-12-19

**Authors:** Sen Yang, Zhi Tian, Yi Feng, Kun Zhang, Yongchun Pan, Yuan Li, Zhichao Wang, Wenhao Wei, Xiaochen Qiao, Ruhao Zhou, Lei Yan, Qian Li, Hua Guo, Jie Yuan, Pengcui Li, Zhi Lv

**Affiliations:** 1 Second Clinical Medical College, Shanxi Medical University, 382 Wuyi Road, Shanxi 030001 Taiyuan, People’s Republic of China; 2 Department of Orthopedics, Shanxi Key Laboratory of Bone and Soft Tissue Injury Repair, The Second Hospital of Shanxi Medical University, 382 Wuyi Road, Shanxi 030001 Taiyuan, People’s Republic of China; 3 Department of Orthopedics, The Second People’s Hospital of Changzhi City, 83 Peace West Street, Shanxi 046000 Changzhi, People’s Republic of China; 4 Department of Orthopedics, The Third people’s Hospital of Datong City, Shanxi 037006 Datong, People’s Republic of China; 5 Shanxi Bethune Hospital, Third Hospital of Shanxi Medical University, Shanxi Academy of Medical Sciences, Tongji Shanxi Hospital, 030032 Taiyuan, People’s Republic of China; 6 Department of Orthopedics, JinZhong Hospital Affiliated to Shanxi Medical University, 689 Huitong South Road, Shanxi 030600 Jinzhong, People’s Republic of China

**Keywords:** Osteosarcoma, Tumor microenvironment, Transcriptomics, Metabolomics

## Abstract

**Background:**

Recently, increasing attention has been drawn to the impact of the tumor microenvironment (TME) on the occurrence and progression of malignant tumors. A variety of 3D culture techniques have been used to simulate TME in vitro. The purpose of this study was to reveal the differences in transcriptional and metabolic levels between osteosarcoma (OS) 2D cells, 3D cells, 3D cell-printed tissue, isolated tissue, and transplanted tumor tissue in vivo.

**Methods:**

We cultured the OS Saos-2 cell line under different culture methods as 2D cells, 3D cells, 3D cell-printed tissue and isolated tissue for 14 days and transplanted tumors in vivo as a control group. Through transcriptomic and metabonomic analyses, we determined the changes in gene expression and metabolites in OS tissues under different culture methods.

**Results:**

At the transcriptional level, 166 differentially expressed genes were found, including the SMAD family, ID family, BMP family and other related genes, and they were enriched in the TGF-β signaling pathway, complement and coagulation cascades, signaling pathways regulating pluripotency of stem cells, Hippo signaling pathway, ferroptosis, cGMP-PKG signaling pathway and other pathways. At the metabolic level, 362 metabolites were significantly changed and enriched in metabolic pathways such as the Fc Epsilon RI signaling pathway, histidine metabolism, primary bile acid biosynthesis, steroid biosynthesis, protein digestion and absorption, ferroptosis, and arachidonic acid metabolism. After integrating the transcriptome and metabolomics data, it was found that 44 metabolic pathways were changed, and the significantly enriched pathways were ferroptosis and pyrimidine metabolism.

**Conclusion:**

Different culture methods affect the gene expression and metabolite generation of OS Saos-2 cells. Moreover, the cell and tissue culture method in vitro cannot completely simulate TME in vivo, and the ferroptosis and pyrimidine metabolism pathways mediate the functional changes of OS Saos-2 cells in different microenvironments.

**Supplementary Information:**

The online version contains supplementary material available at 10.1186/s12920-022-01419-1.

## Introduction

Osteosarcoma (OS) is the primary malignant bone tumor that most commonly affects children, adolescents, and young adults [1]. Due to its easy metastasis and recurrence, the treatment effect is not good. Although today’s standard therapy for OS includes neoadjuvant chemotherapy with doxorubicin, methotrexate and cisplatin followed by surgical resection of the primary tumor and adjuvant chemotherapy [2], the 5-year survival rates for patients with localized disease are between 50% and 70%, but in patients with metastases, the prognosis remains poor (< 20%) [3].

The two-dimensional (2D) culture of tumor cells is being actively used to reveal the complex mechanisms of cancer pathogenesis and the approaches of drug therapy. However, 2D cell culture lacks cell–cell and cell-extracellular matrix interactions. These interactions are critical for the regulation of signaling pathways and gene expression, which is essential for proper cellular function [4]. In recent years, researchers have paid increasing attention to the role of the TME in malignant tumors [5]. The TME comprises various cell types (endothelial cells, fibroblasts, immune cells) and extracellular components (cytokines, growth factors, hormones, extracellular matrix) that surround tumor cells and are nourished by a vascular network [6]. The progression, resistance to drugs, invasion, and metastasis of tumor parenchymal cells are affected by the bidirectional interaction between tumor cells and the TME [7]. Therefore, the analysis of tumor cells alone is not a comprehensive approach to study tumor progression, metastasis, and drug resistance [8]. Only by further understanding and clarifying the mechanism of action between tumor cells and adjacent cells in the TME can the progression of tumor cells be better controlled, and this can provide ideas for the subsequent clinical implementation of effective targeted therapy [9, 10]. To fully simulate the real TME, three-dimensional (3D) cell culture technology was developed in vitro. The 3D cell culture displays physiologically relevant phenotypes, such as cell growth and interactions with its surroundings in a multidimensional structure [11, 12]. Cells grown as 3D cultures exhibit altered cell cycle durations, morphologies (as aggregates or spheroids), susceptibility to drugs, metabolism, and gene and protein expression [13]. It has been found that 2D and 3D primary cell cultures of a different sarcoma subtype have significant differences in gene expression and transcriptome analysis [14]. The 3D cultured method microtissues reflect the tissue heterogeneity of OS and potential suitable tools for drug development toward personalized medicine [3]. As a branch of tissue engineering, cell 3D printing technology is essentially a simulation of the real environment of cell growth. This technology uses computer-aided design (CAD) and computer-aided manufacturing, and it can facilitate the building of a patient’s specific 3D complex living tissue via the direct deposition of biomaterials, biomolecules, and cells [15]. Another technology to simulate the TME in vitro is isolated tumor tissue culture. In a 3D culture system, small pieces of breast cancer tissue were embedded in soft rat collagen I cushions, and after 2–3 weeks of culture, tumor cells spread into the collagen, forming structures similar to those observed in human tumors [17].

Metabolomics has been widely used in cancer metabolism and biomarker identification to infer the onset and progression of cancer [18]. Metabolites, the final products of various biological processes, hold promise as accurate biomarkers that reflect upstream biological events such as genetic mutations and environmental changes [19]. The upstream metabolome can predict the occurrence of a certain biological event from the level of gene expression. Transcriptomic data have been analyzed to identify TME-related prognostic genes in OS [5] and adrenal cortical carcinoma [20]. Integration of transcriptomics and metabolomics may better clarify the biological regulatory mechanism than either approach alone.

In this study, culturing OS using 3D culture, 3D cell-printed tissue and isolated tissue is to effectively simulate the TME in vivo and accurately explore the molecular mechanism of the biological behavior of OS. Light microscopy, live/dead cell staining, and cell counting kit-8 (CCK-8) detection methods confirmed that each 3D tissue was successfully cultured, and then the 2D cells, each 3D cultured tissue and fresh isolated tumor tissue were subjected to transcriptomic and metabolomic detection. After analysis, the changes in gene expression and metabolites of OS cells under different culture methods could be found, and the enriched related signals and metabolic pathways could be clearly identified. Finally, transcriptomic and metabolomic data were integrated to clarify the possible regulatory mechanism specifically affected by different TMEs in OS.

## Materials and methods

### Cell lines and culture

Human OS Saos-2 cell lines were originally obtained from the American Type Culture Collection (Manassas, VA, USA) and cultured in Dulbecco’s modified Eagle’s medium-nutrient mixture F12 (DMEM-F12) with low glucose (Gibco, USA). All culture media were supplemented with 100 units mL^−1^ penicillin, 100 μg mL^−1^ streptomycin, and 10% heat-inactivated fetal bovine serum (FBS, ExCell Bio, China) in a humidified atmosphere of 5% CO_2_ at 37 °C.

### Animal model

Animal experiments were approved by the Animal Care and Use Committee of Shanxi Cancer Hospital (protocol number:2022039). Four-week-old female BALB/c nude mice (n = 6) were from Charles River (Beijing, China), randomized and implanted subcutaneously with 1 × 10^6^ Saos-2 cells in 200 µL Matrigel in their axilla. The dynamic growth of implanted tumors was monitored weekly. At week 2 post inoculation, the mice were euthanized using 5% isoflurane for 3 min and their tumors were dissected. The excised tumor was divided into three parts: one part was used for isolated tissue culture, and the other two parts were used as fresh isolated tumor tissue for transcriptomic and metabolomic detection.

### 3D cells, 3D cell-printed tissue and isolated tissue culture

Matrigel (Corning, NY, USA) dissolved overnight at 4 °C was thoroughly mixed with OS Saos-2 cells at a concentration of 4:1. The final concentration of cells was 1 × 10^6^/ml. After making 3D cells, 50 µl of the mixture was injected into a 60 mm petri dish, and then 5 ml of complete medium was added.

The 3D cell-printed bioink contained 10% w/v methylacrylated gelatin (Shangpu, Beijing, China), 0.5% w/v alginate (Shangpu, Beijing, China), 0.25% w/v blue light initiator (Shangpu, Beijing, China) and 1 × 10^6^/ml OS Saos-2 cell suspension. The configured bioink containing living cells were placed on the bioprinter (Shangpu, Beijing, China). Computer parameters were set: the size of the printing structure was 7 mm × 7 mm × 1 mm, the shape was a three-dimensional grid, the nozzle size was selected to be 200 μm, the printing speed was 4–5 mm/s, the extrusion speed was 0.75–0.85 mm^3^/s, and the temperature of the ink cartridge and the printing platform were set to 18 °C and 4 °C, respectively. The 3D-printed tissue was placed in a 60 mm cell culture dish, after cross-linking with blue light and calcium chloride, 5 ml of complete medium was added.

The in vitro culture of isolated tissue was trimmed with ophthalmic scissors to approximately 2 mm × 1 mm × 1 mm, Matrigel was dissolved overnight at 4 °C, 50 µl was spread on a petri dish, the chopped tissue pieces were placed on Matrigel, and 5 ml of complete medium was added. All tissues were cultured at 37 °C in a humidified atmosphere with 5% CO_2_.

### Live/dead staining

Live/Dead kits (Beibo, Shanghai, China) can evaluate the cell growth state and define the cell growth form. Calcein-AM stains living cells green, and PI stains dead cells red. Saos-2 3D cells, 3D cell-printed tissue, and isolated tissues were cultured for 14 days, and live and dead cells were stained on Days 1, 3, 7, and 14. Each tissue was washed with PBS and stained with Calcein-AM and PI. After 0.5 h of incubation, the stained cells were observed by an inverted fluorescence microscope (Olympus, Japan).

### Cell viability assay

The cell viability of each cultured tissue was measured by CCK-8 (MCE, Shanghai, China) assay. The tissue cultured for 1, 3, 7, and 14 days was washed with PBS, and 1000 µL of DMEM/F12 and 100 µL of CCK-8 working solution were added. After incubation for 3 h, the absorbance was measured by a microplate reader (Molecular Devices, USA) at a wavelength of 450 nm.

### Statistical analysis

Statistical analysis was performed with GraphPad® Prism 8.0. Data are expressed as the mean.

### Transcriptome analysis

OS 2D cells, 3D cells, 3D cell-printed tissue and isolated tissue cultured for 14 days were sampled and immediately frozen in liquid N for storage until RNA extraction and transcriptome analysis. Fresh isolated tumor tissue without culture was used as a control. The experimental process for the transcriptome analysis was conducted by LC-Bio Technology Co., Ltd. (Hangzhou city, China) according to the standard provided by the Illumina Company (San Diego, CA, USA). Total RNA from each tissue sample was isolated using TRIzol reagent (Thermo Fisher, CA, USA). The total RNA quantity and purity were checked using a Bioanalyzer 2100 and RNA 6000 Nano LabChip Kit (Agilent, CA, USA). The mRNA with PolyA was specifically captured by two rounds of purification using Dynabeads Oligo (dT) (Thermo Fisher, CA, USA). After purification, the mRNA fragments were segmented into short fragments using magnesium ions at high temperatures. According to the protocol of the manufacturer’s mRNA-seq sample preparation kit (Illumina, San Diego, CA, USA), the cleaved RNA fragments were reverse-transcribed to produce a cDNA library with a final size of 300 bp ± 50 bp. Finally, we performed paired-end sequencing (PE150) on an Illumina NovaSeq 6000 (LC-Bio Technology Co., Ltd., Hangzhou, China) following the vendor’s recommended protocol.

Cutadapt software was used to filter out disqualified sequences to obtain valid data. After reference genome comparisons of valid data were performed using HISAT2 software, the transcripts were reconstructed using StringTie, and the expression levels of all genes in each sample were calculated. Fragments Per Kilobase Millio (FPKM) values were used to measure gene expression in different samples. DESeq2 software was used to analyze differentially expressed genes (DEGs) selected with the parameter false discovery rate (FDR) < 0.05 and an absolute fold change of ≥ 2. Gene Ontology (GO) and the Kyoto Encyclopedia of Genes and Genomes (KEGG) databases were used to reveal the functional enrichment of DEGs.

### Metabolome analysis

Metabolome analysis was performed by LC-Bio Technology Co., Ltd. (Hangzhou city, China) using the liquid chromatography-tandem mass spectrometry (LC–MS/MS) method. The original sample was taken and ground with liquid N, followed by methanol and acetonitrile. The processed samples were collected according to the machine instructions of the LC–MS system and the high-resolution tandem mass spectrometer TripleTOF 6600 Plus (SCIEX, UK). The liquid phase model (UPLC, SCIEX, UK) was used for chromatographic separation. Reversed-phase separation was performed on an ACQUITY UPLC T3 column (100 mm × 2.1 mm, 1.8 μm, Waters, UK). Compound Discoverer 3.1.0 software was used for data processing, and metabolites were annotated by KEGG and Human Metabolome Database (HMDB). R software Meta X was used for statistical analysis of data and screening of metabolites with significant differences. The R software metaX was used for data statistical analysis and significant difference metabolite screening: the differential metabolites were screened based on the variable important for the projection (VIP) value of partial least squares method-discriminant analysis (PLS-DA) by multivariate analysis, fold change and the *P* value of Student’s t test by univariate analysis. The screening conditions were VIP ≥ 1, fold change ≥ 2 or ≤ 0.5, and *P* ≤ 0.05.

All methods were carried out in accordance with relevant guidelines.

## Results

### OS Saos-2 cells were successfully cultured in each 3D tissue

**Fig. 1:**
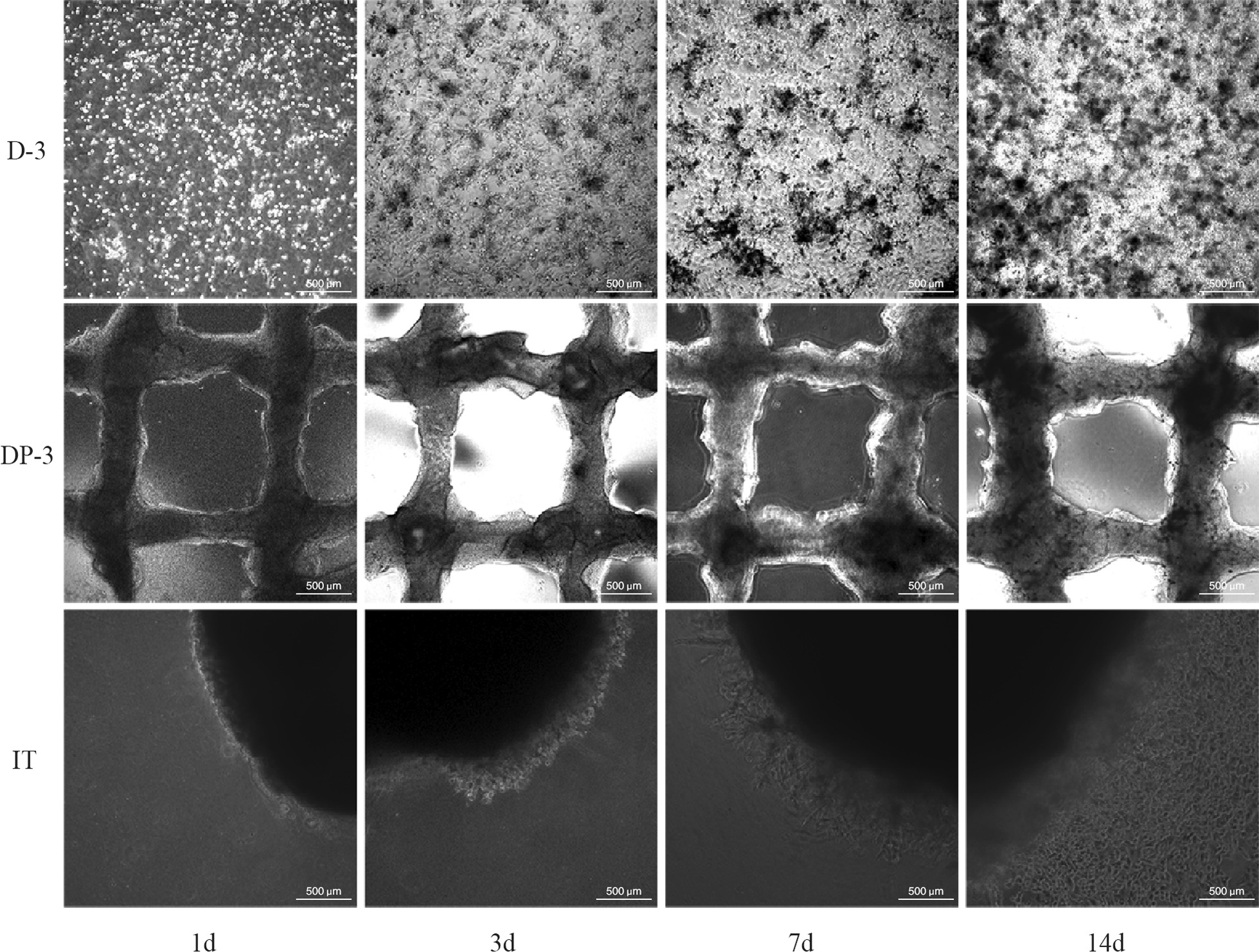
Microscopic bright field of each 3D cultured OS tissue. D-3 is 3D cells. DP-3 is 3D cell-printed tissue. IT is isolated tissue

Each 3D OS tissue was successfully constructed in vitro (Additional file 1: Fig. S1)and cultured for 14 days. The bright field image of the microscope showed that the 3D cells (D-3) were single round cells on Day 1, and the cells began to grow in a 3D cluster after 3 days of culture. On Day 7, 3D cell clusters of different sizes were formed. By Day 14, multiple 3D cell clusters were observed. OS Saos-2 cells in 3D printed tissue (DP-3) were also visible in the bioink with the extension of culture time. In isolated tissue (IT), assisted by Matrigel, low cell growth was observed in the surrounding tissues after one day of culture. On Day 7, obvious radial growth of OS cells was observed; on Day 14, 3D cell fusion was observed in the surrounding tissue (Fig. 1).

**Fig. 2:**
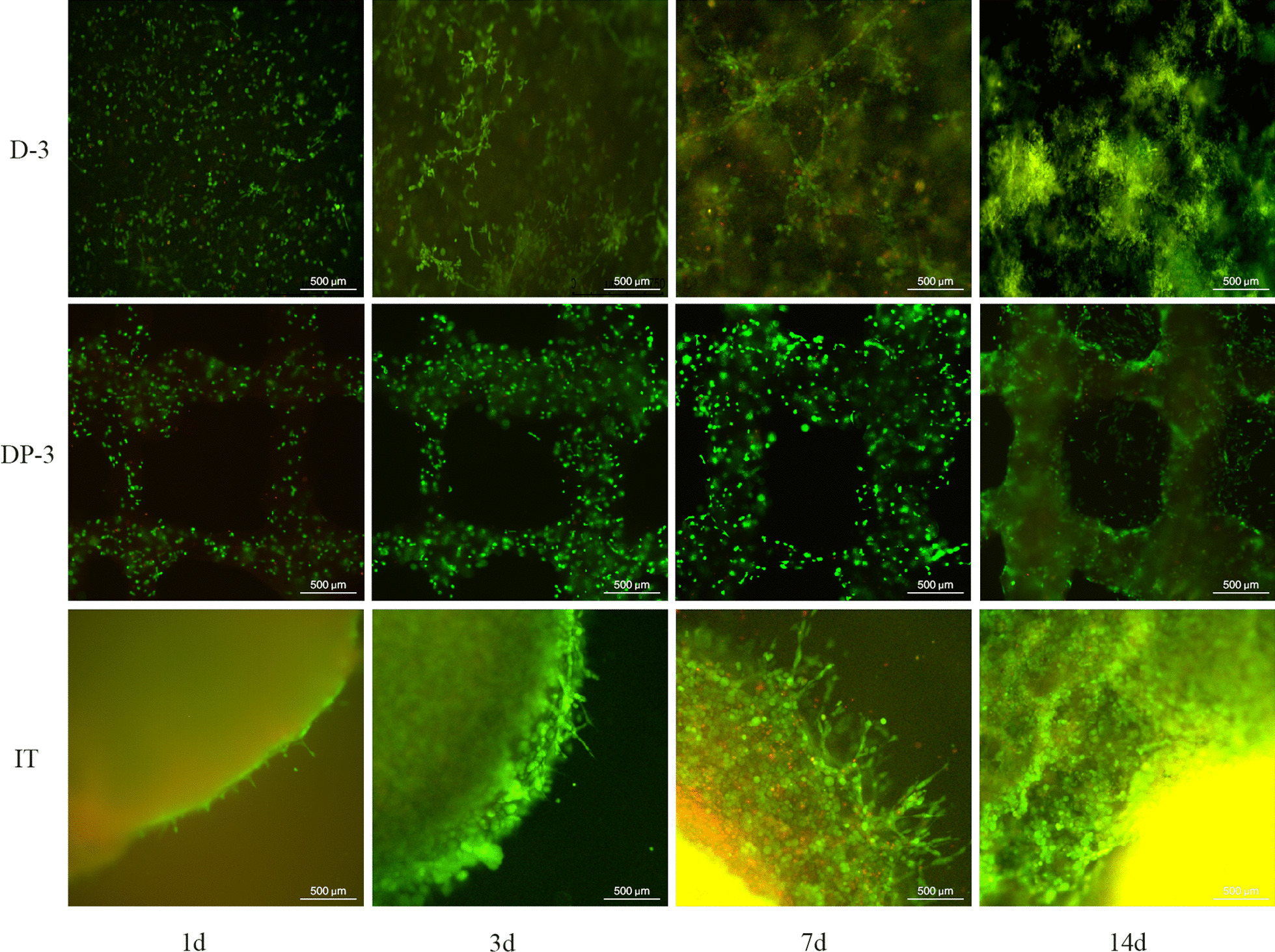
Fluorescence image of Live/dead staining of each 3D cultured OS tissue. Green fluorescence indicates live cells and red fluorescence indicates dead cells

Live/ dead staining can reflect the growth state of cells. The D-3 group grew grape-like on the 3rd day after culture, radiated on the 7th day, and had irregular clumps on the 14th day. With the extension of culture time, single round cells gradually decreased, while irregular small block 3D cells gradually increased in the DP-3 group. After 14 days of culture, OS cells broke through the binding of bioink and obviously grew outward. The 3D cell morphology of OS cultured in the IT group for 14 days was the same as that in the bright field image under the microscope (Fig. 2).

**Fig. 3:**
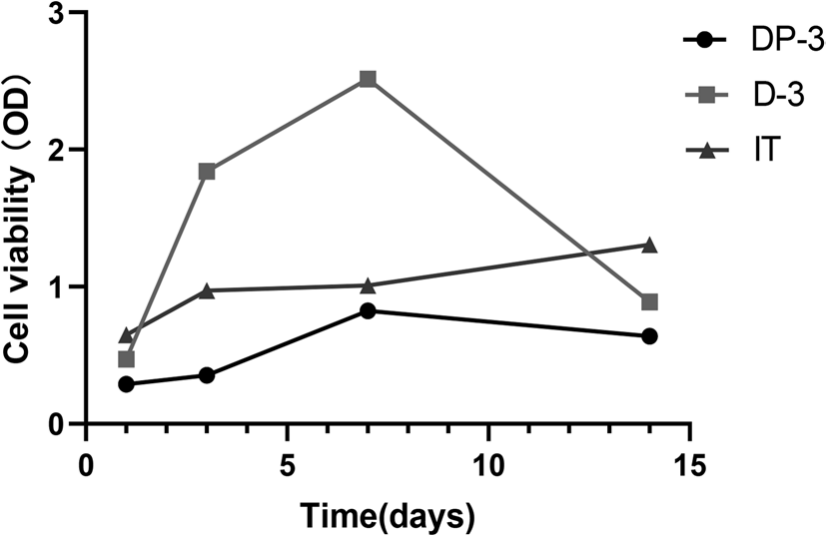
Cell viability of 3D cultured OS tissue at different time periods was detected by CCK-8 assay

To evaluate the cell viability of each 3D cultured OS tissue, the OD value measured by CCK-8 assay was used. The higher the OD value, the better the cell viability. The OD value of IT group increased gradually with the extension of culture time. On the 7th day, the OD value of D-3 group and DP-3 group increased rapidly, while on the 14th day, the OD value of both groups decreased and was lower than IT group (Fig. 3). This figure shows that we successfully cultured 3D tumor tissues in vitro.

### Transcriptomic profiles of OS 2D cells, 3D tissues and fresh isolated tumor tissue

**Fig. 4:**
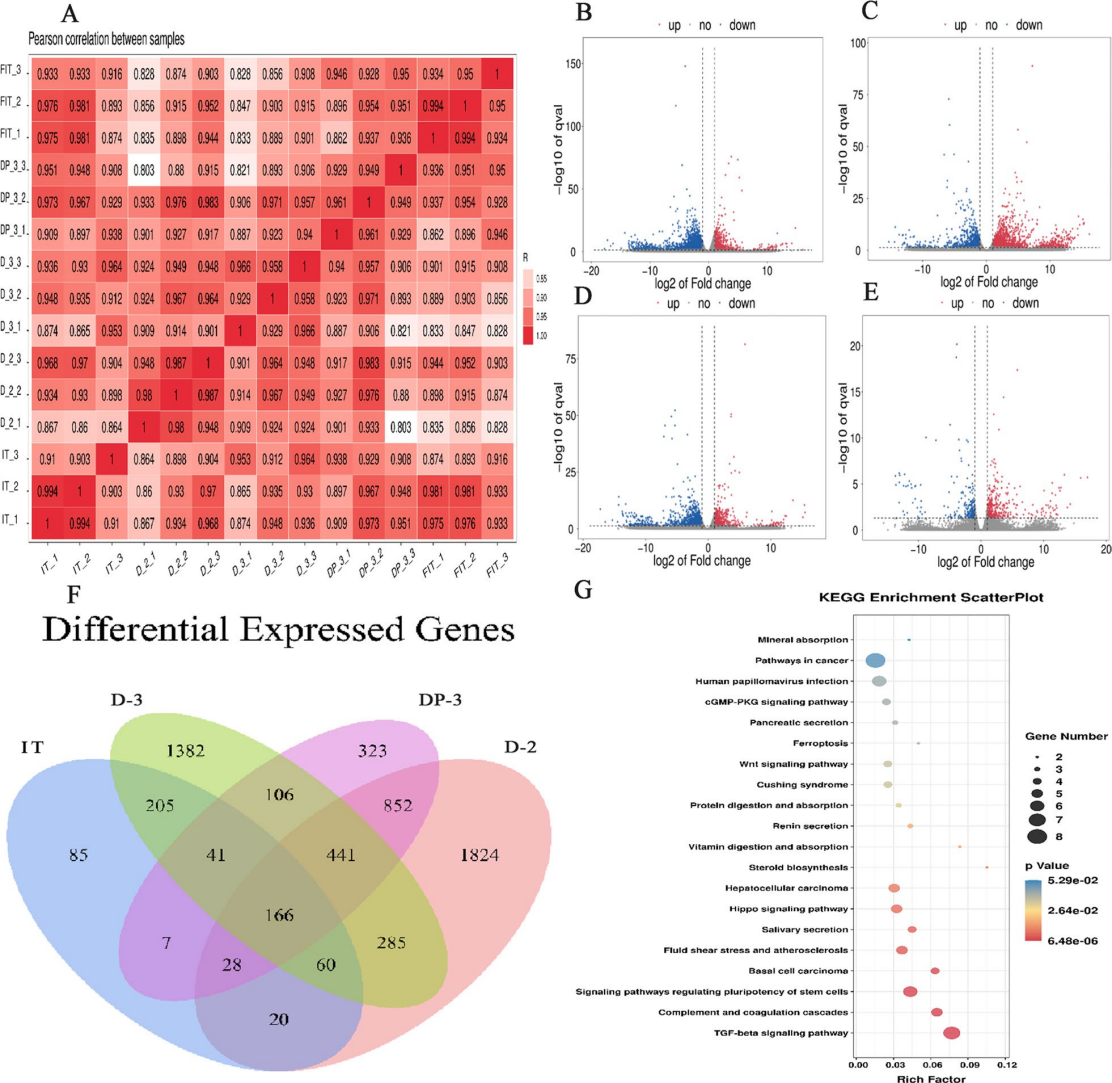
The transcriptome analysis of OS cells and tissues under different culture methods. **A** Heatmap of Repeated Correlations Between Samples. **B–E** The DEGs after comparison between each group were represented by volcano plots. **F** DEGs compared between groups were represented by Venn diagrams. **G** Bubble diagram for KEGG pathway enrichment analysis of DEGs

To explore the changes in the gene transcription level of OS cells in different culture environments, the fresh isolated tumor tissue (FIT) from mouse study, 2D cells (D-2), 3D cells, 3D cell-printed tissue and isolated tissues were analyzed by transcriptome analysis. The RNA sequencing data were first charged, and it was found that the whole process was stable and the data were reliable (Additional file 5: Table S1). Through the correlation analysis of the gene expression of each group of samples, it was found that the correlation coefficients between the samples were all close to 1, indicating that the samples clustered well (Fig. 4A). Compared with the FIT group, 3676 differentially expressed genes (DEGs) were identified in the D-2 group (665 upregulated and 3011 downregulated genes), 2686 DEGs were identified in the D-3 group (1808 upregulated and 878 downregulated genes), 1964 DEGs were identified in the DP-3 group (483 upregulated and 1481 downregulated genes), and 612 DEGs were identified in the IT group (400 upregulated and 212 downregulated genes) (Fig. 4B–E).

After the DEGs screened in each comparison group were analyzed by Venn, 166 common DEGs were identified (Fig. 4F) among which genes related to OS disease were BMP2, BMP4, SMAD6, SMAD9, ID1, ID3, LEFTY1, FZD6, WNT10B, DKK1, NFATC2, HMOX1, FTL and so on (Additional file 6: Table S2). To further identify the major molecular pathways and gene functions, the DEGs were mapped to terms in the KEGG database for gene annotation. The analysis revealed that 144 pathways involved in a wide range of physiological and pathophysiological processes were affected by different culture environments. The top 20 enriched pathways included the TGF-β signaling pathway, complement and coagulation cascades, signaling pathways regulating pluripotency of stem cells, Hippo signaling pathway, ferroptosis, and cGMP-PKG signaling pathway (Fig. 4G). In more detail, some genes related to the TGF-β signaling pathway, including ID1 and ID3 of the ID family and SMAD6 and SMAD9 of the SMAD family, were significantly upregulated. Regarding those in the Hippo signaling pathway, genes belonging to the BMP family were downregulated, such as BMP2 and BMP4. In addition, different culture environments could significantly upregulate ferroptosis signaling pathway-related genes, such as HMOX1 and FTL.

### Metabolomic profiles of OS 2D cells, 3D tissues and fresh isolated tumor tissue

**Fig. 5:**
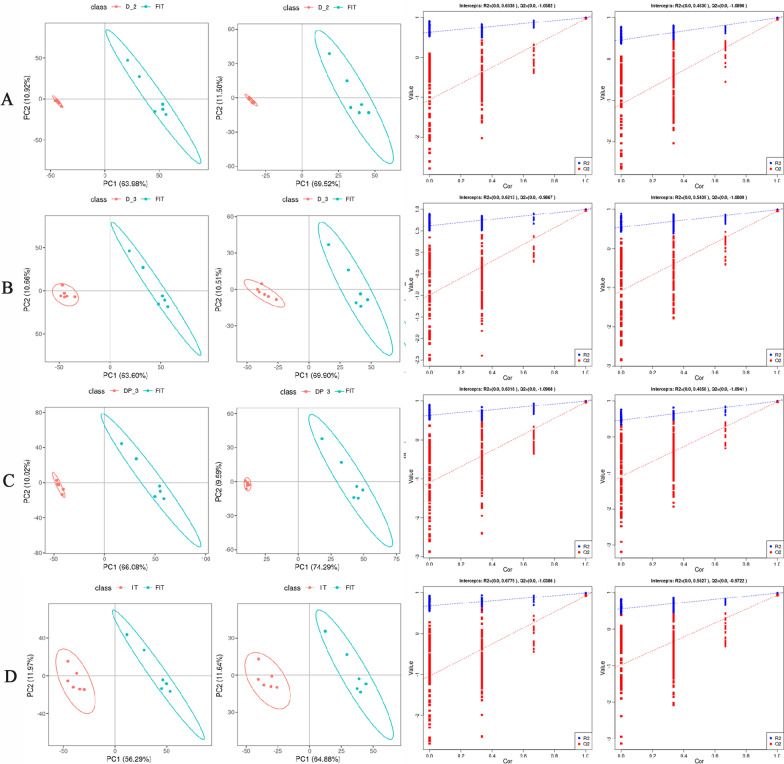
The metabolomic analysis of OS cells and tissue under different culture methods. **A**, **B**, **C**, and **D** represent the classification trend diagram (PLS-DA) and evaluation diagram obtained by comparing the D-2 group, D-3 group, DP-3 group and IT group with the FIT group, respectively. The first two graphs of each group are PLS-DA in positive and negative ion modes, and the last two graphs are 200 times cross validation for the models constructed in both modes

Metabolomics, as a technique for studying the final outcome of a molecular regulatory event, can help us to clarify the differences in metabolic levels of OS cells under different culture methods. After preprocessing of the raw data, multivariate statistical analysis was used to distinguish metabolic differences among the five groups. Data quality control was analyzed by total ion chromatogram (TIC), indicating that the instrument was in good condition and the signal was stable during the entire sample detection and analysis process ( Additional file 2: Fig. S2). The clear separation between these five groups was visually displayed using the principal component analysis (PCA) map in positive (POS) and negative (NEG) ion modes, indicating that different culture environments resulted in obvious biochemical changes in OS Saos-2 cells (Additional file 3: Fig. S3). Further analysis of the constructed PLS-DA model was performed by 200 rounds of cross validation. After analysis, it was found that the Q2 intercepts of POS and NEG between each comparison group were all less than 0, indicating that the established model was objective and authentic. The R2 values in the upper right corner of the figure were all close to 1, indicating that the model had good explanatory ability, while the Q2 values were close to 1, indicating that the model had good predictive ability (Fig. 5).

**Fig. 6:**
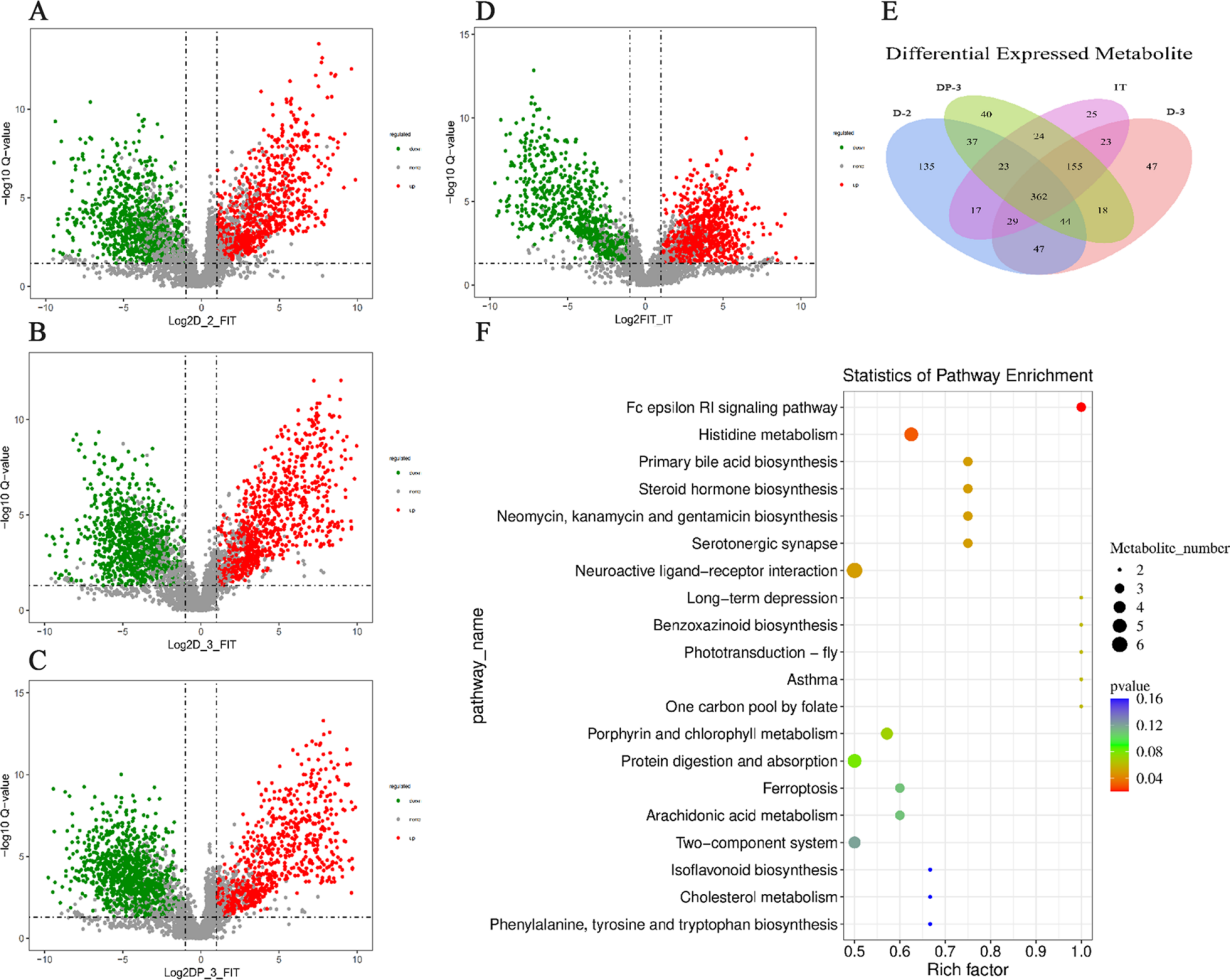
The metabolomic analysis of OS cells and tissues under different culture methods. **A**–**D** The differential metabolites after comparison between each group were represented by volcano plots. **E** The differential metabolites compared between groups were represented by Venn diagrams. **F** Bubble diagram for KEGG pathway enrichment analysis of differential metabolites

The metabolomic profiling analysis indicated that compared to the FIT group, 1723 differential metabolites were annotated in the D-2 group (873 upregulated and 850 downregulated metabolites), 1845 differential metabolites were annotated in the D-3 group (914 upregulated and 931 downregulated metabolites), 1795 differential metabolites were annotated in the DP-3 group (788 upregulated and 1007 downregulated metabolites), and 1633 differential metabolites were annotated in the IT group (800 upregulated and 833 downregulated metabolites) (Fig. 6A–D). The screened differentially metabolized ions were imported into the HMDB database after removing duplicates, and then through Venn analysis, 362 differential metabolites were identified (Fig. 6E). These differential metabolites included arachidonic acid, l-glutamic acid, glutamylcysteine synthetase, histidine, indole, aspartic acid, histamine, pseudouracil nucleoside, 3-urea-propionic acid, cytosine, methylmalonic acid, and 5-methylcytosine. Enrichment analysis of differential metabolites was carried out through the KEGG database, and 161 differential metabolic pathways were finally obtained, including the Fc Epsilon RI signaling pathway, histidine metabolism, primary bile acid biosynthesis, steroid biosynthesis, protein digestion and absorption, ferroptosis, arachidonic acid metabolism, and phenylalanine, tyrosine and tryptophan biosynthesis (Fig. 6F).

### Integrative analysis of transcriptomic and metabonomic results

As described above, many differentially altered transcripts and metabolites enriched in many pathways were identified. To systematically and comprehensively understand the changes in the molecular functions of OS Saos-2 cells under different culture methods, transcriptome and metabolome data were integrated, and Venn analysis was performed (Additional file 4: Fig. S4). As a result, changes were found in 44 pathways, including ferroptosis, protein digestion and absorption, pyrimidine metabolism, glycerophospholipid metabolism, glycolysis/gluconeogenesis, oxidative phosphorylation, cysteine and methionine metabolism (Additional file 7: Table S3). In the ferroptosis pathway, the associated differential genes were HMOX1 and FTL, and the metabolites were arachidonic acid, L-glutamic acid, and gamma-glutamylcysteine. In the pyrimidine metabolic pathway, the associated differential genes were CMPK2, and the metabolites were pseudouridine, 3-ureidopropionic acid cytosine, methylmalonic acid, and 5-methylcytosine. In the glycerophospholipid metabolic pathway, the differentially associated genes were LCAT, and the metabolites were 1-palmitoylglycerophosphocholine, sn-glycero-3-phosphoethanolamine and phosphoethanolamine.

## Discussion

OS is a malignant cancer of bone tissue among adolescents with high morbidity and mortality [21]. Accumulating evidence has claimed that the 5-year survival rate for patients with OS is still low [22]. Although several studies have suggested that dysfunction of bone development and bone remodeling might contribute to OS initiation, the underlying mechanisms remain largely unknown [23, 24]. Therefore, it is very important to clarify the specific mechanism of OS. The concept of the TME has transformed the understanding of tumors from local to global analysis, and the purpose of in vitro 3D tumor cell culture technology is to simulate the real growth environment of tumor cells in vivo. It is possible to induce a TME in cells growing as 3D cultures [13]. In this study, the currently used in vitro 3D cell and tissue culture methods were applied to OS, and it was found that when in vitro 2D or 3D cell and tissue culture methods were compared with in vivo tumor growth, there were significant differences at the transcriptional and metabolite levels. It was clear and undoubted that different culture environments in vitro caused changes in the function of OS Saos-2 cells, which mainly included genes involved in the TGF-β signaling pathway, Hippo signaling pathway, ferroptosis, and signaling pathway regulating stem cell pluripotency. Among them, ferroptosis was more prominent in the holistic analysis, suggesting that it may largely mediate changes in cell function caused by different TME.

Ferroptosis as a form of cellular necrosis was proposed by Dixon in 2012. Unlike autophagy and apoptosis, ferroptosis is an iron- and reactive oxygen species (ROS)-dependent cell death characterized by cytological changes, including reduction or disappearance of mitochondrial ridges, rupture of the mitochondrial outer membrane, and mitochondrial membrane condensation [25–27]; however, the cell nucleus remains intact and is devoid of chromatin clotting, unlike in apoptosis [28, 29]. As an effector molecule of ferroptosis, HMOX1 in the TME has been found to be associated with poor patient prognosis [30–32]. HMOX1 is a 32 kDa protein that predominantly localizes to the endoplasmic reticulum (ER) [33, 34], mitochondria [35], and nucleus [36]. HMOX1 can be expressed in a variety of cells within the TME, including both malignant tumor cells and stromal cell populations, such as macrophages, dendritic cells and regulatory T cells [37]. HMOX1 significantly attenuated erastin-induced ferroptosis in renal epithelial cells [38]. BAY11–7085 induced ferroptosis of breast cancer cells by upregulating HMOX1 [39]. Overexpression of HMOX1 can significantly inhibit the proliferation of OS U2OS cells [40]. In fact, HMOX1 has dual roles in cancer cells. Low expression of HMOX1 have cytoprotective effects by scavenging ROS after activation, and excessive activation can increase ROS levels and the iron concentration to induce apoptosis [41]. In addition to the significant upregulation of HMOX1, ferritin light chain (FTL) was also altered when we analyzed differentially expressed genes. FTL is a unique functional component of ferritin that is overexpressed and plays an important role in a variety of human malignancies. For example, FTL protein is significantly higher in gastric cancer than in paracancerous tissues [42]. In HeLa cells and glioblastoma cells, the expression of FTL can promote the growth of cancer cells [43, 44]. It has been suggested that high expression of FTL may characterize a more aggressive type of malignancy with an increased proliferation capacity [45]. In this study, we found high expression of FTL in Saos-2 OS cells under different culture methods in vitro, indicating that the proliferation and invasion ability of tumor cells cultured in vitro are stronger than those in vivo, which also conforms to the theory that tumor cells cultured in vivo are slower than those in vitro.

Pyrimidine metabolism integrates nucleoside recovery, de novo nucleotide synthesis and catalytic degradation [47, 48], which is a key pathway for DNA replication and RNA synthesis. Current studies have found that some genes of pyrimidine metabolism are involved in epithelial-mesenchymal transformation (EMT) function in cancer and are associated with morphologic and functional epithelial-like phenotypic loss of solid cancer, increased resistance to treatment, and enhanced stem-cell-like characteristics of cancer cells [49]. There are few studies on pyrimidine metabolites, which are only found to accumulate in and out of cells, but their functions are not described in detail. Recent studies on pancreatic cancer have shown that the TME can be rich in pyrimidines secreted by nontumor entities such as tumor-associated macrophages and stromal astrocytes [50], where macrophages can secrete a wider range of pyrimidines, including thymine, cytosine, cytosine, dUMP, uridine and uracil, with thymine being the most secreted [51]. However, our study found that the content of cytosine in 2D cells and 3D cultured tissues outside OS was lower than that in vivo, which may be because in vivo nontumor cells can also secrete pyrimidine metabolites, thus affecting the biological behavior of Saos-2 cell replication.

In this study, the combined analysis of transcriptomics and metabolomics provides a broad perspective on the functional changes of OS Saos-2 cells under different TMEs. However, there are some limitations of this study. First, we cultured only 3D cells and tissues in vitro for 14 days, possibly ignoring the effect of time on cell function. Therefore, we can further clarify the changes in genes and metabolites of OS cells at different time points after culture. Second, the changes in transcripts need further validation by some molecular biology analytical techniques, such as real-time polymerase chain reaction and western blot.

## Conclusion

Compared with tumor growth in vivo, 2D cells, 3D cells, 3D cell-printed tissue and isolated OS tissue were significantly different. According to the gene transcription level, 166 genes were significantly changed in OS Saos-2 cells under different microenvironments and were enriched in the TGF-β signaling pathway, complement and coagulation cascades, signaling pathways regulating pluripotency of stem cells, the Hippo signaling pathway, ferroptosis, and the cGMP-PKG signaling pathway. At the metabolic level, 362 metabolites with significant changes were found to be enriched in the Fc Epsilon RI signaling pathway, histidine metabolism, primary bile acid biosynthesis, steroid biosynthesis, protein digestion and absorption, ferroptosis, arachidonic acid metabolism, and phenylalanine, tyrosine and tryptophan biosynthesis. Importantly, a comprehensive analysis of transcriptional and metabolic outcomes revealed that ferroptosis and pyrimidine metabolic pathways primarily mediate functional changes in OS Saos-2 cells in different microenvironments. To our knowledge, this is the first study combining transcriptional and metabolic profiling to analyze the functional mechanisms underlying alterations in OS Saos-2 cells in different TMEs.

## Supplementary Information

**Additional file 1**: Fig S1. Appearance of each 3D cultured OS tissue. **A** 3D cells, **B** 3D cell-printed tissue, **C** isolated tissue.

**Additional file 2**: Fig S2. Total ion Chromatogram of OS samples based on ionic strength. The signal intensity of the sample quality spectrum can be controlled as a whole by taking the time point as the abscissa and the sum of the intensity of all ions in the mass spectrum as the ordinate. **A** POS, **B** NEG.

**Additional file 3**: Fig S3. Principal component analysis of ion quantification in each 3D OS tissue. QC samples are clustered, indicating that the experimental data is of high quality. **A** POS, **B** NEG.

**Additional file 4**: Fig S4. Number of pathways obtained after combined transcriptome with metabolomics analysis.

**Additional file 5**: Table S1. RNA-Seq data quality assessment. Valid Ratio refers to the proportion of valid reads. Q30% is the proportion of bases with a quality value ≥ 30, which should normally be greater than 85%. GC content% is the proportion of GC content, which should normally be between 40% and 50%.

**Additional file 6**: Table S2. 166 differentially expressed genes were analyzed by Veen.

**Additional file 7**: Table S3. Pathway information from combined transcriptome with metabolomics analysis.

## Data Availability

The raw sequence data generated in the current study are available in the NCBI Sequence Read Archive (accession number: GSE201050).

## Abbreviations

TME: Tumor microenvironment
OS: Osteosarcoma
2D: Two-dimensional
3D: Three-dimensional
IT: Isolated tissue
FIT: Fresh isolated tumor tissue
CCK-8: Cell counting kit-8
DMEM-F12: Dulbecco’s modified Eagle’s medium-nutrient mixture F12
FPKM: Fragments Per Kilobase Millio
DEGs: Differentially expressed genes
KEGG: Kyoto Encyclopedia of Genes and Genomes
HMDB: Human Metabolome Database
PLS-DA: Partial Least Squares Method-Discriminant Analysis
HMOX1: Heme oxygenase1
FTL: Ferritin light chain.
