# Supplementary figures and images for: Transcriptomics and metabolomics reveal changes in the regulatory mechanisms of osteosarcoma under different culture methods in vitro
Source: BMC Med Genomics. 2022 Dec 19;15:265. doi: 10.1186/s12920-022-01419-1 (PMC9762085; doi:10.1186/s12920-022-01419-1)

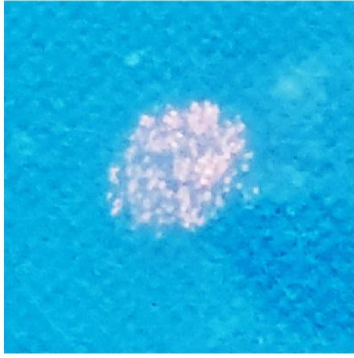

A

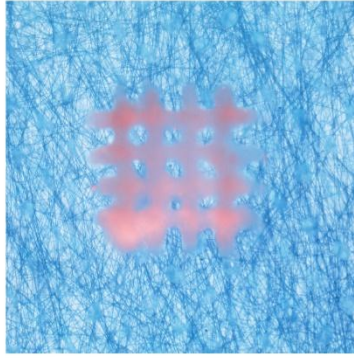

B

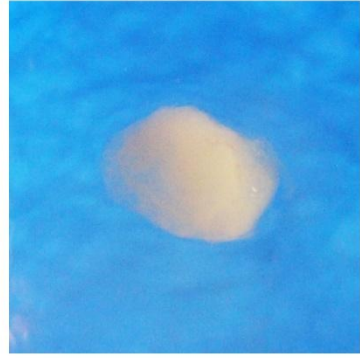

C

Supplement: Supplementary file 1 — Additional file 1: Fig S1. Appearance of each 3D cultured OS tissue. A 3D cells, B 3D cell-printed tissue, C isolated tissue. [file 12920_2022_1419_MOESM1_ESM.pdf]

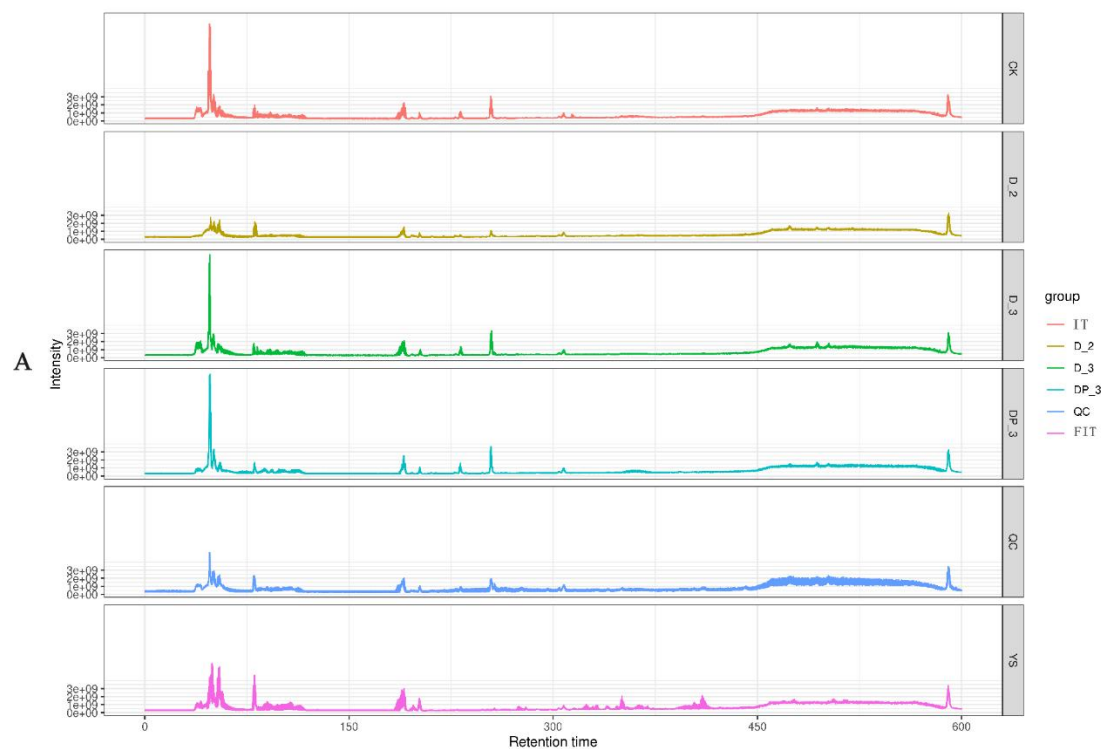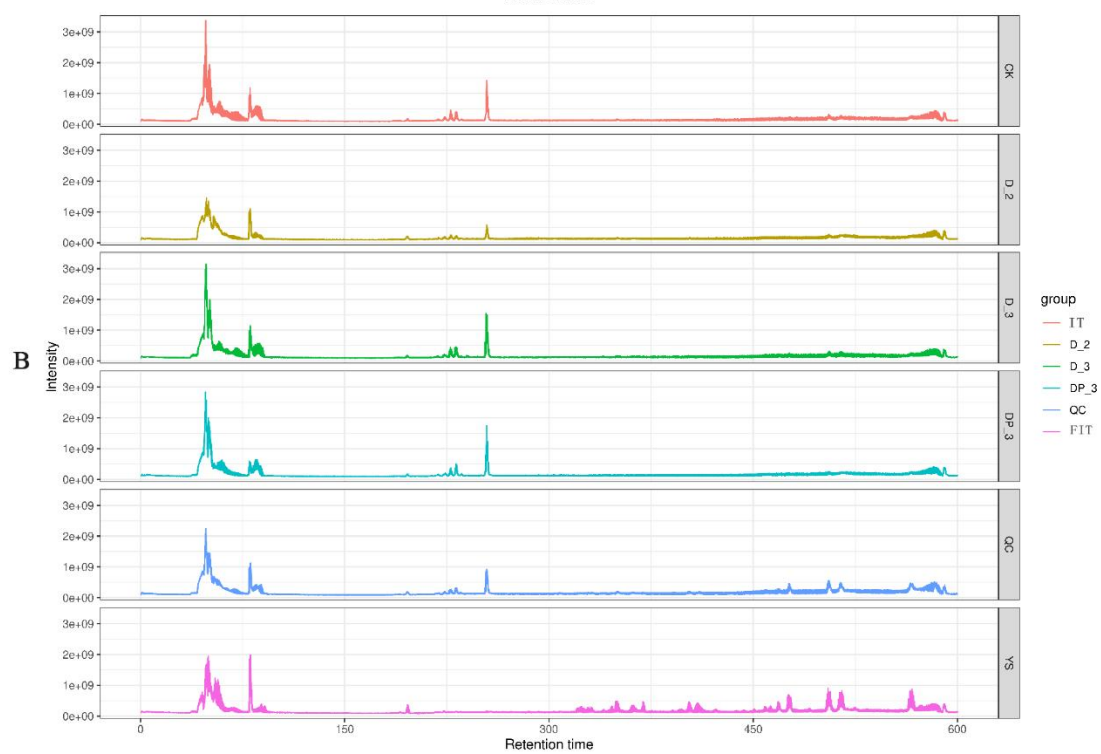

Supplement: Supplementary file 2 — Additional file 2: Fig S2. Total ion Chromatogram of OS samples based on ionic strength. The signal intensity of the sample quality spectrum can be controlled as a whole by taking the time point as the abscissa and the sum of the intensity of all ions in the mass spectrum as the ordinate. A POS, B NEG. [file 12920_2022_1419_MOESM2_ESM.pdf]

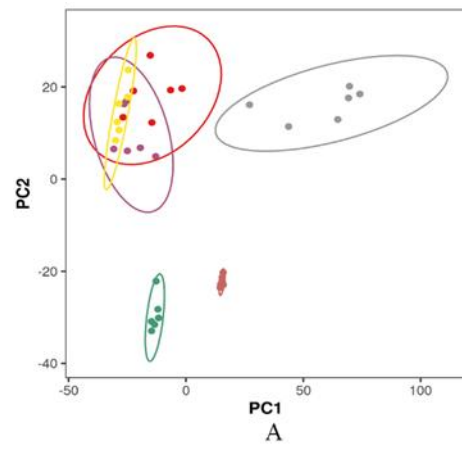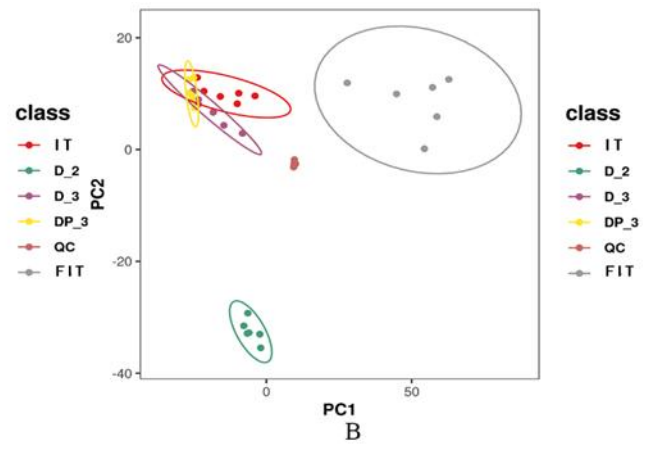

Supplement: Supplementary file 3 — Additional file 3: Fig S3. Principal component analysis of ion quantification in each 3D OS tissue. QC samples are clustered, indicating that the experimental data is of high quality. A POS, B NEG. [file 12920_2022_1419_MOESM3_ESM.pdf]

Differential Expressed Pathway

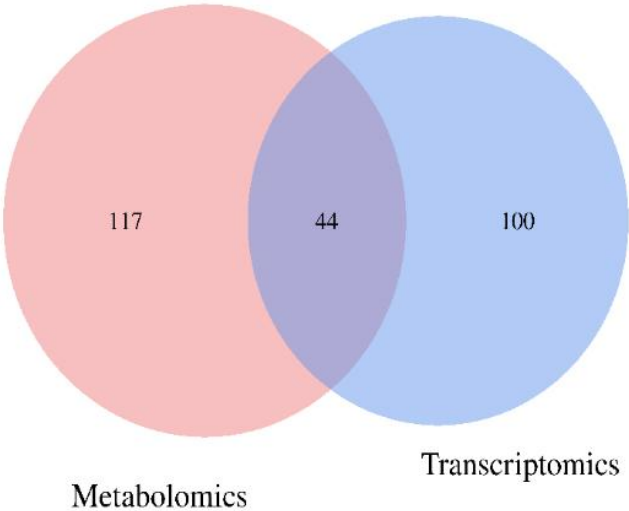

Supplement: Supplementary file 4 — Additional file 4: Fig S4. Number of pathways obtained after combined transcriptome with metabolomics analysis. [file 12920_2022_1419_MOESM4_ESM.pdf]
